# From Predefined Auditory Stimuli to Brain-Guided Generative Auditory Neuroscience

**DOI:** 10.3390/biology15212006

**Published:** 2026-11-05

**Authors:** Qiang Li, Jianmei He

**Affiliations:** College of Education Science, Guizhou Education University, Guiyang 550018, China; hejianmei@gznc.edu.cn

**Keywords:** brain-guided generation, auditory neuroscience, generative models, auditory representations, adaptive experimental design

## Abstract

Auditory cognitive neuroscience has moved from controlled sounds toward increasingly complex stimuli, but the choice of what sounds to test remains largely determined before neural responses are observed. A complementary experimental logic is emerging, in which neural responses are used not only to evaluate predefined stimuli but also to guide the generation and selection of subsequent stimuli. Here, we articulate a perspective on brain-guided generative auditory neuroscience, in which human neural responses are related to manipulable auditory representations to establish brain–sound relationships that guide which stimuli are generated or selected for subsequent testing. This framework enables the investigation of auditory structures that may not be specified in advance, including response-relevant stimulus organization, representational boundaries, contextual dependencies, and individual- or state-dependent brain–sound variation. We further discuss technical routes for establishing brain–sound relationships and using them to guide stimulus generation, as well as how iterative cycles of generation and human testing can progressively refine these relationships through recursive brain–sound inference. The value of this approach depends not on producing unusual sounds, but on whether brain-derived guidance yields reproducible insights beyond predefined or non-neural alternatives.

## Simple Summary

Auditory neuroscience has moved from simple controlled sounds toward increasingly complex and naturalistic auditory experiences. However, researchers usually decide which sounds to test before observing how the brain responds. This Perspective develops brain-guided generative auditory neuroscience, a framework in which neural responses help guide the generation and selection of future auditory stimuli. By linking brain activity with flexible sound representations, this approach may reveal previously inaccessible aspects of auditory organization and enable new ways to investigate how the brain represents sounds. Rather than replacing conventional auditory experiments, brain-guided generation provides a complementary strategy in which neural evidence contributes to shaping the next steps of scientific discovery.

## 1. Introduction

Auditory cognitive neuroscience has followed a related trajectory, progressively broadening the range and complexity of auditory stimuli brought under experimental study [1,2]. Early experimental work frequently relied on tones, simple sound complexes, isolated syllables, and other parametrically controlled stimuli, whose acoustic properties could be manipulated with precision while unwanted variation was minimized [1]. This degree of experimental control provided a powerful basis for isolating specific dimensions of auditory processing, but it also confined investigation to a relatively narrow region of the stimulus space encountered in everyday listening [3]. Over time, the experimental repertoire expanded to include speech, music, continuous narratives, and other structured and naturalistic sounds that engage auditory and cognitive processes not readily captured by simpler paradigms [4,5]. With this shift, the ecological and cognitive scope of auditory research broadened accordingly, allowing researchers to investigate auditory processing under conditions that more closely reflect the acoustic and scene complexity, temporal dynamics, contextual structure, and behavioral demands of real-world listening.

This expansion has changed what can be measured, but it has not by itself changed how information typically flows through the experiment. Whether the stimulus set consists of pure tones or hours of natural speech, investigators generally define the candidate sounds, contrasts, or sampling strategy before the neural responses that constitute the primary evidence are observed. Those responses are then used to characterize how the auditory system represents the selected stimuli and dimensions. Naturalistic paradigms therefore expand the territory available for investigation, but they do not necessarily make neural evidence an active determinant of where that investigation proceeds next. The unresolved opportunity is not simply to enlarge the auditory stimulus repertoire, but to use what has been learned from the brain to help determine the next direction of experimental inquiry.

A different experimental logic emerges when neural evidence is used to guide what stimulus structures are constructed and how subsequent experiments are designed. Several elements relevant to such a logic have appeared before in auditory research, although in different contexts and for different purposes. For example, neural responses have been used to influence stimulus construction itself: cortical responses have informed the construction of receptive-field-matched sounds and the adaptive search of multidimensional acoustic spaces [6,7,8]. Related work has explored another relationship between brain activity and auditory output, including early brain–computer music interfaces in which EEG features steered generative musical rules [9]. Decoding and reconstruction studies, meanwhile, approached the brain–sound relationship from yet another direction, establishing that information about speech and music can be recovered from human neural activity [10,11,12,13]. Taken together, these examples illustrate some of the ways in which neural information has already entered the relationship between brain and sound—not only as a measured response, but also in the construction, control, or recovery of auditory information.

Beyond these auditory precedents, related principles of neural-guided stimulus exploration have emerged across neuroscience. In visual neuroscience, generative models have been combined with neural response measurements to search high-dimensional stimulus spaces and identify synthetic stimuli that evoke targeted neural activity, including approaches that optimize artificial images toward responses of individual neurons or cortical regions [14,15,16,17]. In animal sensory neuroscience, adaptive stimulus optimization and closed-loop neurophysiological paradigms have used neural responses to guide stimulus selection or modification, extending earlier receptive-field mapping approaches toward more efficient exploration of high-dimensional stimulus spaces [6,7,8,18,19]. Although these approaches differ in modality and experimental implementation, they share a common principle: neural activity can function not only as an outcome measure for evaluating stimuli, but also as a source of information for determining which stimuli should be generated, selected, or tested next.

Recent methodological advances have made it increasingly feasible to connect these previously separate operations within the same experimental process. Methods for relating complex sounds to neural responses can provide an operational link between brain-derived response structure and stimulus variation [20,21], while generative audio systems can instantiate candidate sounds within flexible stimulus spaces [22,23], and optimization or adaptive-design procedures can help direct which parts of those spaces are explored [19]. Their relevance to brain-guided generation lies in how these functions can be combined, when needed, to translate neural evidence into manipulable auditory interventions, so that what is learned from the brain can influence what sounds are constructed and tested next. A recent study by Xing et al. provides a particularly instructive example of such integration in audition [24]. The authors combined neuroimaging, a deep neural network, brain-based sound synthesis, and psychophysics to identify synthetic sounds associated with speech- and music-preferring auditory regions; critically, the generated sounds were then replayed and shown to elicit category-consistent cortical and behavioral responses despite being acoustically unlike natural exemplars. The importance of the study lies in the way several of these operations are linked within a single auditory experiment: brain-derived representations inform the construction of novel sounds, and the resulting stimuli are then evaluated through new neural and behavioral measurements, thereby completing a full experimental cycle from neural representation to stimulus intervention and back to empirical evaluation. The acoustic divergence of the synthesized sounds from natural speech and music is particularly informative: despite this divergence, they elicited category-consistent neural and behavioral responses, suggesting that brain-guided synthesis may provide a means of probing aspects of auditory representation that are not readily revealed by natural exemplars alone.

By showing that experimentally informative stimuli need not remain confined to acoustic forms defined in advance, Xing et al. point to a broader role for neural information in revealing stimulus structure that was not specified beforehand and translating that structure into subsequent experimental interventions. We use the term brain-guided generative auditory neuroscience to describe a broader experimental framework built around this principle, in which brain-derived responses or representational relationships are connected to manipulable aspects of sound and used to shape what stimuli are generated or selected; the resulting stimuli are evaluated through new neural or behavioral measurements, and the resulting evidence informs scientific questions, models, or subsequent experimental decisions. The broader conceptual shift is toward an experimental logic in which brain-derived information becomes an active part of experimental design, helping determine what stimulus structures, transformations, contexts, or distinctions should be constructed and tested as inquiry proceeds.

**Figure 1:**
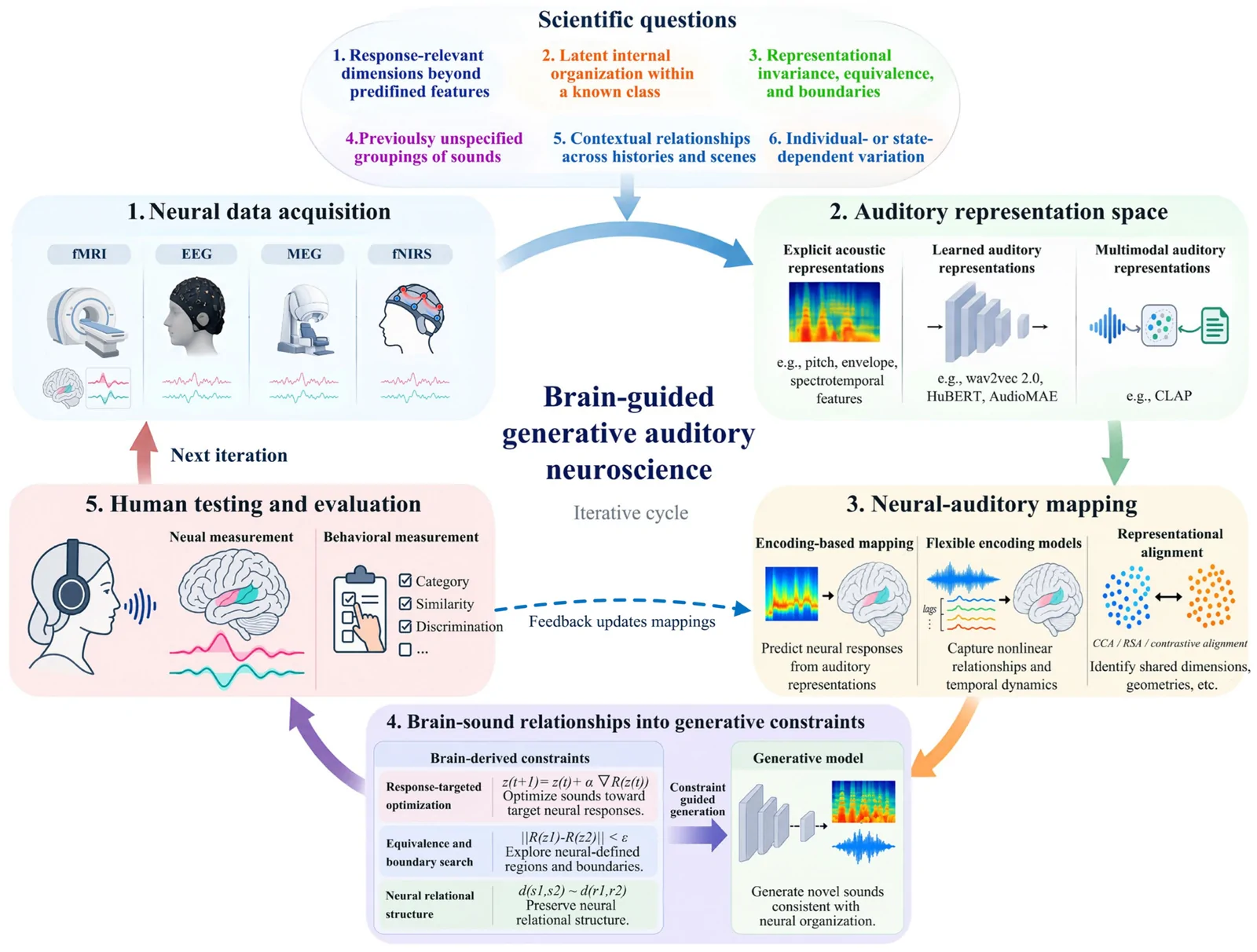
Overview of brain-guided generative auditory neuroscience. Neural measurements, auditory representations, and brain–sound mappings are integrated to derive generative constraints, producing new sounds that are evaluated through human neural and behavioral measurements. The resulting measurements feed back to refine brain–sound mappings, enabling iterative refinement across successive cycles of generation and evaluation.

In this Perspective, we synthesize these converging developments into a framework for brain-guided generative auditory neuroscience by proposing six scientific questions that organize the principal opportunities for brain-guided generative auditory experimentation and by extending the generation-and-testing cycle into recursive brain–sound inference, in which new human measurements progressively refine brain–sound relationships and guide subsequent stimulus construction. We then examine the technical routes through which neural measurements, auditory representations, brain–sound mappings, and generative constraints can be combined to translate brain-derived information into experimentally testable sounds. Finally, we consider how the added value of brain guidance can be evaluated against matched predefined or non-neural alternatives, together with the limits that govern the interpretation of the resulting discoveries. An overview of this brain-guided generative auditory neuroscience framework is provided in Figure 1, which summarizes how scientific questions, human neural measurements, auditory representations, brain–sound mappings, and generative constraints are integrated into an iterative cycle of stimulus discovery and evaluation. The key terms used throughout this Perspective and their distinct roles are summarized in Table 1.

## 2. Scientific Questions and Experimental Strategies for Brain-Guided Generative Auditory Experimentation

The scientific value of brain-guided generative auditory neuroscience lies not simply in producing a larger or more realistic stimulus repertoire, but in using brain–sound relationships to determine which stimulus structures should be constructed and tested. This capability enables experiments to explore response-relevant dimensions, representations, and relationships that may remain inaccessible through predefined stimulus designs. On this basis, we propose six classes of scientific question for which brain-guided generation may provide particular experimental leverage: identifying response-relevant stimulus dimensions beyond predefined acoustic features; revealing latent organization within auditory representations; testing representational invariance, equivalence, and boundaries through controlled stimulus variation; discovering previously unspecified neural-response-defined groupings of sounds; examining how contextual and relational structure shapes auditory representations; and characterizing how stimulus–brain relationships vary across individuals and internal states.

### 2.1. Identifying Discriminative Structure Underlying Known Auditory Distinctions Beyond Predefined Dimensions

Auditory perception and cognition often distinguish between sound classes even when the stimulus structure supporting that distinction is not obvious. Speech provides an intuitive example: listeners readily distinguish speech from other complex vocal sounds, yet the relevant distinction may not be reducible to familiar acoustic dimensions such as pitch, intensity, or spectral content. The critical structure may instead involve combinations of multiple properties, higher-order patterns of stimulus variation that are not captured by conventional acoustic descriptions, or directions that become apparent only within richer representations of sound. Conventional experiments can test a candidate dimension once it has been specified, but they are less suited to cases in which the form of the relevant stimulus variation is itself unknown. In such cases, the scientific problem is not whether the two classes can be distinguished, but what structure in the sounds supports that distinction. Brain-guided generation provides a way to use neural responses associated with the known distinction to identify candidate stimulus determinants that were not specified beforehand and then construct new sounds through which those determinants can be tested experimentally.

A practical requirement is therefore a sufficiently expressive and manipulable stimulus space in which complex sound variation can be explored without restricting the experiment to a small set of predefined acoustic axes. Recent brain-guided auditory studies provide one concrete implementation of this strategy: sounds can be represented within a high-dimensional auditory model, and measured cortical responses can be related to variation in that representation to guide synthesis of novel sounds associated with target neural responses [24]. The relevant stimulus directions were therefore not selected in advance as familiar acoustic variables, illustrating how neural evidence can identify variation within a richer auditory space that warrants subsequent experimental testing.

### 2.2. Revealing Latent Internal Organization Within Predefined Auditory Classes

A further opportunity for generative auditory neuroscience is to reveal potential organizational patterns of auditory perception that are not captured by established auditory categories. Conventional experiments often begin by defining categories or contrasts and then examine how the brain distinguishes them. Yet even categories long established in auditory research may conceal finer patterns of organization among the sounds they bring together. Even when exemplars are treated as equivalent with respect to the properties that define the established category, variation in their neural responses may reveal systematic relationships among them. The resulting organization may appear as relatively discrete groupings, continuous gradients, graded transitions, or more complex arrangements across stimulus–neural space. Brain-guided generation can then relate this emerging response structure to a sufficiently rich and manipulable representation of sound and construct new stimuli to test whether the inferred organization extends beyond the original exemplars and reflects a reproducible feature of auditory representation.

### 2.3. Revealing Representational Invariance, Equivalence, and Boundaries

A third distinctive use of generative auditory neuroscience is to reveal the range of stimulus variation that a given neural representation treats as equivalent and where that equivalence breaks down. Conventional experiments can test invariance when plausible stimulus dimensions or transformations can be specified in advance—for example, by varying speaker identity, sound level, or another candidate property and asking whether the neural representation remains stable. The more difficult case arises when investigators do not know which stimulus dimensions, combinations, or ranges define that equivalence in the first place, particularly when the relevant variation may lie along higher-order directions that are not readily expressed in familiar acoustic terms. Brain-guided generation provides a way to approach this problem by using neural responses themselves as the criterion for defining candidate equivalence. Rather than assuming in advance which acoustic transformations should preserve a representation, this approach can begin with sounds that reliably evoke a target neural response and identify new sounds whose neural consequences remain consistent with that response. In this sense, the objective is not simply to generate more examples of a known sound class, but to determine the range of stimulus variation that remains compatible with a particular neural representation. This question is central to auditory invariance: the same speech content or auditory object can be realized by acoustically different signals—for example, when it is produced by different speakers, presented at different levels, or embedded in different contexts—yet it may still be recognized as the same underlying source or category. Speaker normalization in human auditory cortex provides one example of such tolerance to acoustic variation [25]. Brain-guided generation extends this principle by allowing the relevant range of preserved representations to be explored beyond transformations chosen by experimenters in advance. It can therefore reveal whether a neural representation corresponds to a broad region of tolerated variation, a relatively restricted region, or a transition between distinct representational states.

### 2.4. Discovering Previously Unspecified Auditory Groupings Through Brain-Guided Generation

A fourth possibility is to use brain-guided generation to discover previously unspecified organizations or groupings of sounds that emerge from their relationship with neural responses. Conventional auditory studies generally begin with at least a coarse organization of the stimulus set—for example, speech, music, vocalizations, or environmental sounds—and examine neural variation within or between those predefined domains. A more exploratory strategy would begin instead with a broad and heterogeneous collection of sounds and ask whether reproducible relationships between stimulus structure and neural responses reveal coherent groupings or organizations that are not captured by conventional stimulus categories. The sounds forming such a grouping need not share an obvious semantic label or differ from other sounds along a familiar acoustic dimension. Their common structure may instead involve higher-order configurations or combinations of stimulus variation that become apparent only within a sufficiently expressive representation of sound. Unlike the preceding approach, which asks how broadly an established neural representation extends across stimulus variation, the aim here is to determine whether previously unspecified organizations of auditory space can emerge from the joint structure of neural responses and stimulus variation. Such neural-response-defined groupings can reveal latent organizations of auditory space that are not captured by conventional stimulus taxonomies and can provide new hypotheses about how sounds are represented beyond experimenter-defined categories. Whether these groupings correspond to perceptual categories or meaningful auditory classes, however, remains a separate question that requires direct behavioral validation.

### 2.5. Discovering Previously Unspecified Response-Relevant Contextual Relationships Across Auditory Histories and Scenes

A fifth distinctive use of generative auditory neuroscience is to discover response-relevant relationships across auditory histories and scenes that are difficult to define or identify in advance. Auditory responses are shaped not only by the immediate acoustic input, but also by what has occurred before it and by the other sources with which it is heard [26,27]. Natural speech, music, and environmental recordings contain rich and intertwined relationships among auditory events, preceding context, background structure, and concurrent sources, making it difficult to isolate which aspects or combinations of these relationships systematically shape neural responses [28]. Conventional experiments can test contextual influences when a plausible factor can be specified beforehand, but they are less suited to situations in which the relevant structure is distributed across multiple interacting relations and its effective form is itself uncertain. Brain-guided generation offers a way to approach this problem by sampling diverse combinations of sounds, histories, and scenes, relating the resulting neural variation to a sufficiently expressive representation of their relational structure, and identifying candidate patterns that repeatedly covary with neural responses across different auditory events. These patterns may correspond to familiar contextual factors, but they may also reflect higher-order combinations or directions within a relational space that are not apparent from any single contextual variable. The aim is therefore not simply to manipulate background or context around a given sound, but to determine which relationships among auditory events, histories, and sources provide reproducible structure for human neural responses.

### 2.6. Probing Individual- and State-Dependent Brain–Sound Relationships

Brain-guided generation also creates an opportunity to move beyond neural organization defined at the group level. Human auditory responses vary across individuals in their magnitude, timing, spectral characteristics, multivariate response structure, and, where spatial information is available, their anatomical distribution or functional selectivity. A stimulus dimension or combination that is strongly associated with a particular neural response in one listener may therefore carry a different weight or neural signature in another. Individual-specific experiments could relate each participant’s responses across a diverse sound set to a manipulable auditory representation and use the resulting correspondence to guide stimulus construction for that listener. Generated sounds could then test whether stimulus dimensions, combinations, or regions of auditory space that are informative for one individual reproduce the same neural effects in others, or instead reveal listener-specific patterns of auditory sensitivity. A useful precedent comes from vision, where synthetic images derived from personalized encoding models elicited stronger responses than images derived from group-level or other-participant models in several higher-order regions [29]. Such comparisons could help distinguish auditory structure that is broadly shared across listeners from structure whose neural expression is substantially individualized.

Individualization may also extend from relatively stable differences between listeners to fluctuations within the same listener over time. Ongoing attention, arousal, affective state, or neural dynamics may change which aspects of a sound are most strongly expressed in the measured response, so the auditory dimensions that are informative for a given listener need not remain fixed from moment to moment. Individualized closed-loop auditory stimulation already provides a relevant precedent: Harlow et al., for example, used each participant’s alpha frequency and auditory ERP latency to time sound presentation according to ongoing EEG activity [30]. In a generative setting, state-sensitive neural measurements could instead be used to determine which parts of an individually calibrated auditory space should be explored or emphasized at a particular moment. This would make it possible to construct stimuli that are more diagnostic of transient changes in attention or auditory sensitivity, and potentially to adapt auditory content as neural responses indicate changing preference or engagement. Real-time neural measures have already been used to update personalized music selection, illustrating how listener-specific brain signals can inform auditory choices as experience unfolds [31]. Extending this logic from selection to generation could allow auditory content to be shaped jointly by relatively stable individual neural characteristics and the listener’s current state.

### 2.7. Scientific Questions for Which Brain-Guided Generation Provides Particular Leverage

The six opportunities considered above differ in their immediate scientific targets, but they converge on a shared scientific rationale for using brain-guided generation. Conventional parametric experiments are most effective when the relevant stimulus dimension, transformation, or relationship can be specified in advance and manipulated systematically. Across Section 2.1, Section 2.2, Section 2.3, Section 2.4 and Section 2.5, however, a central part of the scientific problem is precisely that the response-relevant organization of the stimulus space is not yet known well enough to define the appropriate manipulation beforehand. The unresolved structure takes different forms across these cases: the dimensions or higher-order feature combinations that support an established distinction (Section 2.1), latent organization within an established auditory class (Section 2.2), the extent and boundaries of stimulus variation compatible with a neural representation (Section 2.3), previously unspecified neural-response-defined groupings within auditory space (Section 2.4), and contextual or relational dependencies that do not reduce to a predefined variable (Section 2.5). Taken together, these sections define a common class of scientific question in which the dimensions, boundaries, groupings, or relationships that would ordinarily organize the experiment are themselves objects of inquiry. Brain-guided generation is particularly well suited to this class of question because it allows the experimental space itself to be shaped by brain–sound relationships, with neural evidence guiding where subsequent stimulus generation and testing proceed as the relevant structure becomes clearer.

Section 2.6 introduces a complementary class of question in which the brain–sound relationship itself may vary across listeners or internal states. A stimulus dimension, combination, or region that is strongly associated with a neural response in one listener may carry a different weight or neural signature in another, and the same relationship may change as the listener’s internal state changes. The central question is therefore not only which brain–sound relationships can be identified, but which are shared across listeners, which are individual-specific, and how they change with internal state. Brain-guided generation is particularly well suited to this question because the relationship used to guide stimulus generation need not be fixed at the group level: it can be estimated for an individual, compared across listeners, and updated as internal state changes, making the generality and conditionality of brain–sound relationships experimentally accessible.

These two classes of scientific question define the particular leverage of brain-guided generation: first, situations in which the stimulus structure needed to address the scientific question cannot be fully specified in advance; and second, situations in which the generality and stability of a brain–sound relationship across listeners or internal states is itself under investigation. Table 2 summarizes the specific scientific question, brain-guided generative strategy, and principal insight associated with each of the six opportunities developed in Section 2.1, Section 2.2, Section 2.3, Section 2.4, Section 2.5 and Section 2.6.

## 3. Technical Routes for Brain-Guided Generative Auditory Neuroscience

The scientific strategies described above do not depend on any single neural recording technique, auditory model, or generative architecture. Their implementation requires choices about the neural signal, auditory representation, brain–sound mapping, and generative constraint, with different combinations suited to different scientific questions. Section 3.1, Section 3.2, Section 3.3 and Section 3.4 consider these components in turn.

### 3.1. Selecting Neural Signals for Brain Guidance

The neural signal used for guidance should be chosen according to the response structure of interest. fMRI is particularly suitable when the target is a regional response, multivoxel pattern, or distributed representational structure, as demonstrated by studies using activation-targeted stimulus search and neural-guided synthesis across sensory modalities [24,29,32]. EEG and MEG provide complementary access to millisecond-scale temporal dynamics, making event-related activity, oscillatory responses, stimulus tracking, and multivariate temporal patterns potential guidance signals. Importantly, recent work has already linked non-invasive MEG and EEG activity during speech perception to high-dimensional representations from wav2vec 2.0, demonstrating that temporally resolved neural signals can be aligned with learned auditory spaces [33]. fNIRS could provide a slower but portable hemodynamic route when regional cortical responses are sufficient, particularly in naturalistic or device-compatible settings, whereas ECoG and other intracranial recordings provide unusually detailed local spectrotemporal information and have supported reconstruction of perceived speech and music from human auditory cortex [10,11,12]. These methods therefore need not provide the same type of brain guidance: the target may be response magnitude, temporal trajectory, spatial pattern, representational similarity, or another reproducible neural quantity appropriate to the scientific question.

### 3.2. Defining Auditory Representation Spaces for Stimulus Generation

A second choice concerns how sound variation is represented. Interpretable acoustic spaces based on spectrotemporal modulation, envelope, pitch, or related descriptors provide explicit coordinates that can be manipulated and tested directly [34]. Learned auditory models provide a broader alternative when response-relevant structure cannot be adequately captured by such predefined dimensions. Task-optimized auditory networks, self-supervised speech models such as wav2vec 2.0 and HuBERT, and general-audio representations such as AudioMAE provide high-dimensional learned representations in which complex sound structure can be expressed without requiring the relevant acoustic dimensions to be specified in advance [21,35,36,37]. Multimodal audio–language models provide another way to represent sound variation. CLAP, for example, maps sounds and natural-language descriptions into a shared embedding space, providing a high-dimensional representation in which sounds are organized in relation to linguistic descriptions and categories [38]. Rather than assuming that one representation is universally optimal, the choice among acoustic, learned, and multimodal spaces should depend on what forms of sound variation need to be made accessible and manipulable for the scientific question at hand.

### 3.3. Establishing Brain–Sound Relationships

With a candidate auditory space defined, the next technical step is to establish how variation within that space relates to the neural quantity of interest. Several mapping strategies could serve different purposes. A direct route is forward encoding, in which an auditory representation is used to predict the neural response of interest. One recent auditory implementation used regularized linear encoding models to map learned sound representations onto responses in speech- and music-preferring auditory regions and then optimized the input representation through the fitted model to generate response-associated sounds [24]. Such mappings need not be linear: nonlinear encoding functions could be used when neural responses depend on more complex combinations of stimulus features, as demonstrated in human visual fMRI [39]. For temporally resolved EEG, MEG, or intracranial signals, these mappings could additionally incorporate temporal lags or response trajectories. A complementary route is to relate the two spaces at the level of their multivariate structure rather than through one-way prediction. Canonical correlation analysis, representational similarity analysis, and contrastive alignment can identify shared dimensions, geometries, or correspondences between neural and auditory representations [33,40,41]. Together, these approaches provide different ways of identifying the aspects of auditory space that are linked to the neural target, thereby establishing the brain–sound relationship required for subsequent stimulus construction.

### 3.4. Translating Brain–Sound Relationships into Generative Constraints

The establishment of a brain–sound relationship marks the transition from identifying response-relevant structure to determining how that structure can constrain generation while still allowing the resulting stimuli to generalize beyond the sounds used to infer it. Existing generative approaches typically address this problem by defining a controllable stimulus representation space or latent space and conditioning generation on variables that specify the desired properties of the output. For example, latent diffusion models generate diverse auditory signals by sampling within learned latent spaces while conditioning the synthesis process on representations of the intended sound characteristics [22]. Similarly, domain-specific generative systems constrain generation through structured inputs, such as textual, acoustic, or temporal conditions, allowing new instances to be produced while preserving selected properties of the input space [42,43]. In a brain-guided setting, the conditioning information would instead be derived from the established neural–sound relationship: the generator would not simply reproduce previously observed stimuli, but would be constrained to produce novel sounds whose locations in the stimulus space remain consistent with the neural organization being investigated. Such constraints may take different forms depending on the scientific objective, including optimization toward a predicted neural response, exploration of regions defined by neural equivalence criteria, or generation of stimulus sets that preserve or manipulate relationships inferred from neural organization. Similar principles have been explored in visual neuroscience, where neural responses have been used to optimize or constrain generated images, demonstrating that neural-guided generation can serve as a general strategy for stimulus discovery beyond audition [14,15,16,17].

However, brain-guided generation need not be limited to maximizing a single response value. Another possibility is to use the brain-derived relationship to define a region of stimulus space rather than a single optimal point. For example, when the objective is to identify the range of sounds that preserve a target neural representation, the generation process can be constrained by an estimated equivalence region. Active level-set estimation and Bayesian adaptive sampling provide relevant precedents for identifying regions of an input space that satisfy a response criterion while directing measurements toward uncertain boundaries [44,45]. In a brain-guided auditory setting, such approaches could guide the generation of sounds within, near, or beyond a neural-defined region, allowing experiments to test not only whether a response can be elicited, but also where that response remains stable and where it transitions into another representational state. When the brain–sound relationship is defined at the level of representational structure, the inferred relationship can instead be translated into constraints for generating stimulus sets with controlled relational properties. Rather than optimizing only for a high predicted neural response, generation can be used to construct novel sounds that systematically vary along the dimensions predicted to be relevant to the neural representation, while preserving or manipulating specific relationships among stimuli. Similar strategies have been demonstrated in human vision, where neural-network-based image synthesis has been used to generate stimulus sets with controlled representational structures, allowing researchers to test how predefined changes in stimulus relationships are reflected in brain activity [46,47].

However, the analogy with visual neuroscience has important modality-specific limits. Sound is an unfolding signal: its perceptual and neural effects depend on temporal envelopes, fine timing, rhythm, pitch and prosodic trajectories, and relationships between preceding and current events. A sequence of locally plausible segments can therefore remain globally incoherent, especially when speech or music depends on long-range temporal structure [5,26,34]. Auditory objects are also organized through temporal coherence among acoustic features and successive events; disrupting the timing or continuity that links components of a source can alter stream segregation and neural responses even when individual segments remain recognizable [48]. Ecological validity consequently depends not only on waveform fidelity but also, when relevant to the hypothesis, on context, event order, source continuity, and interactions among concurrent sources [2,4,28]. Work on long-form music generation further illustrates that maintaining global structure over extended durations can require explicit modeling of longer temporal contexts, rather than optimizing local audio quality alone [49]. These demands create a distinctive trade-off for brain-guided generation: the generator must preserve task-relevant temporal and contextual relationships while allowing the feature combinations or boundaries under investigation to vary. The appropriate balance is therefore question-dependent: some experiments require coherent naturalistic scenes, whereas others may deliberately use acoustically unusual sounds to isolate a neural distinction. Temporal continuity, perceptual coherence, and ecological validity should be treated as explicit, task-dependent evaluation dimensions, rather than assumed to follow from waveform quality or a high predicted neural response.

Together, these approaches illustrate that brain-guided generation does not correspond to a single optimization strategy, but to different ways of translating neural evidence into constraints on stimulus construction. Depending on the scientific question, the relevant constraint may define an optimal stimulus, a region of equivalent stimuli, a relational structure among stimuli, or a broader organization within auditory space. By converting these inferred structures into experimentally controllable sound variations, generative systems can create stimulus sets that test which aspects of auditory organization are preserved, transformed, or disrupted by specific manipulations.

## 4. A Worked Example: Identifying Unknown Stimulus Dimensions Underlying a Known Auditory Distinction

The framework developed in this Perspective can be illustrated by following a single auditory question from its initial formulation to stimulus generation, human validation, and interpretation. Consider an established auditory distinction, such as the distinction between speech and other complex vocal sounds. The existence of such a distinction is well established, but the stimulus organization supporting it is not necessarily defined by a small number of acoustic variables that can be manipulated in advance. Instead, the relevant structure may depend on higher-order combinations of features, latent directions within an auditory representation, or relationships among stimulus properties that are difficult to specify before neural measurements are considered.

The experiment would begin by sampling neural responses across a sufficiently diverse auditory stimulus space. The discovery stimulus set would include speech, non-speech vocal sounds, environmental sounds, and acoustically diverse intermediate or transformed sounds, providing broad variation across auditory properties rather than restricting the analysis to a small set of predefined acoustic contrasts. Such a stimulus space allows brain–sound relationships to reveal candidate response-relevant structures that may not be apparent from the original stimulus categories or from individual acoustic features considered in isolation.

The choice of neural measurement determines what form of brain-derived information can be used to guide stimulus construction. Spatially resolved measurements such as functional MRI provide neural signals across voxels and regions, from which brain–sound relationships can be derived using individual signals, combinations of sources, or weighted patterns across multiple neural locations. Temporally resolved approaches such as EEG or MEG provide complementary information when the timing of neural responses is central, whereas intracranial recordings such as ECoG offer an alternative when fine-grained local signals are most informative. To illustrate one concrete implementation, consider an fMRI-based experiment in which neural signals extracted from the diverse stimulus set above are used to establish a brain–sound mapping that guides subsequent stimulus generation.

After neural responses have been acquired, the next step is to define the neural signal that will serve as the target for brain–sound mapping. Consider the case in which the experiment aims to identify the stimulus organization supporting the distinction between speech and other complex vocal sounds. Neural responses elicited by the diverse stimulus set would first be characterized to determine how variation across sounds is reflected in activity patterns within the auditory system. These patterns can then be summarized as a brain-derived signal that captures the neural organization relevant to the distinction, for example through multivariate response patterns or weighted combinations of neural signals across informative sources. The resulting neural signal provides a quantitative description of how sounds are represented within the measured system and can be related to variation within an auditory stimulus space to identify candidate dimensions, directions, or regions for subsequent stimulus generation.

The next step is to define an auditory representation in which the diversity of sounds can be expressed and systematically explored. The appropriate representation depends on the type of stimulus structure that may underlie the auditory distinction. When the relevant organization is expected to correspond to interpretable acoustic properties, explicit acoustic representations based on spectral, temporal, or prosodic features provide directly manipulable dimensions for stimulus variation [34]. When the relevant structure is unlikely to be captured by predefined acoustic variables, learned auditory representations offer a broader space in which complex patterns of sound variation can be represented. Such representations can be derived from task-optimized auditory models that capture behaviorally relevant sound features [21], self-supervised speech models such as wav2vec 2.0 and HuBERT that learn hierarchical representations from large-scale speech data [35,36], or general audio models such as AudioMAE that capture broader regularities across sound categories [37]. Multimodal audio representations, such as CLAP, provide another possibility when auditory structure is investigated in relation to semantic or cross-modal information [38]. For this example, a pretrained general auditory representation such as AudioMAE would provide an appropriate starting point because it can capture complex patterns across diverse sounds without requiring the relevant stimulus dimensions to be specified in advance.

The next step is to estimate how variation within the auditory representation corresponds to variation in the neural signal associated with the distinction of interest. A direct approach is to fit encoding models that estimate a mapping between stimulus representations and neural responses. In this framework, the auditory representation serves as the input space, and the estimated parameters describe how different dimensions or combinations of dimensions contribute to the observed neural signal. Regularized linear encoding models provide an interpretable approach by estimating weighted relationships between high-dimensional auditory features and neural responses while controlling model complexity [20,21]. Such models can identify response-predictive directions within the auditory representation space by determining which patterns of stimulus variation are associated with systematic changes in neural activity. When the relevant organization depends on nonlinear interactions among stimulus properties, nonlinear encoding models provide a more flexible alternative [39]. Other approaches, including representational similarity analysis, canonical correlation analysis, and contrastive alignment methods, can identify corresponding structure between neural and auditory spaces without requiring a single predefined predictive direction [33,40,41].

Once the relationship between auditory representations and neural responses has been established, the next challenge is to determine which structures within the stimulus space account for the observed distinction. In this example, the goal is not simply to predict whether a sound will evoke a speech-related response, but to identify the dimensions, combinations of features, or boundaries in auditory space that separate speech from other complex vocal sounds. One approach is to examine the parameters of the learned brain–sound mapping as a representation of discriminative structure. For example, in a linear encoding model, the weight vector linking auditory features to the neural response difference between speech and vocal sounds defines a direction in the auditory representation along which stimulus variation is most strongly associated with the neural distinction. Movement along this direction can therefore reveal whether the distinction depends on a single interpretable property or on a distributed combination of latent features within the auditory space [20,21]. When the relevant organization is not well described by a single direction, multivariate approaches can instead characterize distributed stimulus structures or response-defined boundaries within the auditory space. Classification-based methods provide one possible implementation by identifying boundaries that separate regions associated with different neural organizations, whereas geometry-based approaches such as representational similarity analysis can reveal whether these distinctions correspond to discrete separation, graded transitions, or overlapping structure [20,28]. The resulting boundary can then be examined to determine whether the speech–vocal distinction reflects a relatively sharp transition, a graded continuum, or overlapping regions within stimulus space. Such analyses transform the neural difference between two established categories into experimentally testable hypotheses about the stimulus structures that support the distinction.

The final step is to generate new sounds that directly test the inferred stimulus organization and to use their responses to refine the brain–sound relationship. Sounds can be synthesized by sampling, extending, or perturbing the response-relevant dimensions, combinations, or boundaries identified from the neural mapping, rather than by simply reproducing the original discovery stimuli. For example, if the analysis identifies a latent direction separating speech and other vocal sounds, newly generated sounds can be placed along this direction to determine whether the neural distinction changes gradually, abruptly, or remains stable across intermediate regions of the auditory space. These generated sounds would then be returned to human participants for independent neural and behavioral measurements, providing evidence that supports or challenges whether the inferred stimulus organization captures a genuine property of auditory representation. Crucially, demonstrating that generated sounds are informative is not sufficient to establish that brain guidance was responsible for identifying the stimulus organization. In this example, the contribution of neural guidance could be tested by comparing the same generative and search procedure under matched alternative constraints. For instance, one control condition could use predefined acoustic dimensions or a non-neural objective derived from the auditory representation itself, while another could replace the neural target with shuffled or otherwise mismatched neural information. If brain-guided generation reveals a more reliable speech–vocal transition, a more diagnostic boundary, or stronger generalization to new human measurements than these matched alternatives, this would provide evidence that the neural constraint contributed information beyond the generative procedure alone. Conversely, if similar structures emerge without neural guidance, the interpretation that brain-derived information provided additional scientific leverage would be weakened. Through this iterative process, brain-guided generation becomes a framework in which candidate stimulus structures are discovered, tested, and refined through successive cycles of human measurement.

## 5. From Brain-Guided Generation to Recursive Brain–Sound Inference

Many of the questions opened by brain-guided generation are unlikely to be resolved by a single generation-and-testing step. A candidate feature combination may drive a response without yet defining the limits of the underlying selectivity; a putative representational boundary may be only coarsely localized; and a neurally inferred organization of auditory space may require repeated probing before it can be distinguished from an idiosyncrasy of the initial mapping. In each case, the response to a generated stimulus provides new evidence about which stimulus relationships remain plausible, where a boundary may lie, or which explanation still needs to be distinguished. We refer to the use of this new evidence to update subsequent stimulus construction as recursive brain–sound inference. Its scientific value lies in progressively resolving an uncertain auditory structure or explanation across successive human measurements.

Operationally, each new set of neural measurements can determine which aspect of the inferred stimulus structure should be probed next. Differences among responses to generated sounds can indicate whether a candidate direction should be extended, narrowed, perturbed, recombined, or redirected toward an alternative organization. Subsequent stimuli are then constructed from what the brain has newly distinguished, so that successive measurements progressively refine the structure under investigation. Recursive generation therefore remains brain-guided because the direction of stimulus modification is updated from neural evidence rather than following a fixed acoustic search axis.

Importantly, brain-guided generation does not necessarily require a fully online closed-loop implementation. The temporal dynamics of the recursive cycle depend strongly on the neural measurement modality and the latency between acquisition, modeling, generation, and validation. Electrophysiological methods such as EEG and MEG provide millisecond-scale measurements and are therefore better suited for rapid adaptive paradigms in which neural responses can influence subsequent stimulus selection within relatively short time windows. By contrast, hemodynamic methods such as fMRI offer high spatial resolution and access to distributed representational structure but involve slower physiological dynamics and more complex acquisition constraints, making rapid stimulus-by-stimulus updating less practical. Accordingly, the framework proposed here should initially be understood primarily as an offline or cross-session recursive process: neural measurements inform generative models, generated sounds are subsequently evaluated in humans, and new evidence updates the inferred brain–sound relationship across iterations. Future advances in real-time analysis, accelerated acquisition, and multimodal integration may enable faster online implementations, but such real-time closed loops are not a prerequisite for the scientific logic of brain-guided generation.

Repeated cycles can eventually do more than refine which aspect of a neurally inferred stimulus structure should be probed next. The neural–stimulus relationship that initiates the search is itself inferred from a finite set of sounds and response variation, whereas successive generations may progressively enter combinations, directions, or transitions that were poorly represented in those initial data. If several rounds of human testing reveal systematic response patterns that the original relationship does not capture, those discrepancies become evidence that the relationship itself may be incomplete. Rather than re-estimating it after every generation, the accumulated measurements could periodically be used to revise the correspondence between neural responses and stimulus variation, potentially exposing new directions or dependencies that were not apparent in the initial experiment. The revised relationship might preserve the original stimulus direction, shift it, reveal that several dimensions contribute jointly, or expose a different organization that could not be identified from the initial measurements. Subsequent generation would then proceed from this updated neural–stimulus correspondence, allowing recursion to operate not only on which sounds are constructed next but, when sufficient new evidence has accumulated, on the brain-derived relationship that determines how stimulus variation is constructed in the first place.

These recursive ambitions should be distinguished from related auditory systems that already use neural signals to control or adapt auditory output. More broadly, passive brain–computer interface research has established a basis for adapting human–machine interaction to continuously decoded cognitive or affective states [50]. Within auditory brain–computer interfaces, EEG-based auditory attention decoding has been investigated as a basis for neurosteered hearing devices, including methods designed to adapt to listener-specific conditions [51,52]. More recently, a real-time system based on intracranial EEG dynamically amplified the attended speaker in multi-talker environments, improving speech intelligibility and reducing listening effort [53]. These studies demonstrate that neural information can guide auditory processing in a closed loop. Their primary objective, however, is to recover or enhance a behaviorally relevant sound stream, rather than to generate stimuli for identifying previously unspecified auditory dimensions, locating representational boundaries, or distinguishing competing explanations of auditory organization.

Related principles have also been explored in neuroadaptive media, music-based feedback, and rehabilitation. In music applications, EEG-derived measures have been investigated for personalizing music recommendations [54], while closed-loop systems have used continuously synthesized music to reflect and modulate listeners’ affective states [55]. More recent work has extended this approach to personalized playlists updated using in-ear EEG, demonstrating that neural information can influence music selection and subjective listening outcomes [56]. A systematic review of music-based neurofeedback documents a growing range of experimental approaches while identifying inconsistencies in proposed mechanisms and outcome measures [57]. Rehabilitation-oriented auditory feedback provides another relevant precedent: the Encephalophone has been investigated as an EEG-controlled musical interface, including a clinical pilot study involving people with acquired motor disabilities [58,59]. A broader review of adaptive closed-loop brain–computer interfaces in neurorehabilitation further contextualizes these developments within functional training and recovery [60]. Collectively, these studies establish important precedents for neural decoding, adaptive auditory output, personalization, and feedback. They do not, however, establish that generated sounds can be used to discover previously unspecified auditory organization through iterative refinement of brain–sound mappings. This distinction makes the additional scientific value of brain guidance, rather than merely its ability to control auditory output, the central question for the evaluation framework developed in the following section.

## 6. Evaluating and Implementing Brain-Guided Discovery

The flexibility that gives brain-guided generation its experimental power also creates a central problem of attribution. A generated sound may elicit an unusual neural response, reveal an apparent boundary, or separate two competing explanations, yet the existence of such an effect does not by itself establish that brain-derived information was responsible for finding it. The generator restricts which sounds can be produced, the stimulus representation determines which variations are accessible, a predictive model may favor particular regions of that space, and an adaptive search procedure can locate extreme examples even when its objective contains little specifically neural information. The evidential question is therefore whether brain guidance contributes information that changes what can be discovered beyond what the same generative and search machinery would have produced without that guidance. A brain-guided claim becomes scientifically testable when the experiment specifies an appropriate alternative source of guidance and asks whether the neural information leads to different, more diagnostic, or more reproducible experimental outcomes.

The most direct way to test whether brain guidance adds scientific value is to compare it with matched alternative ways of choosing stimuli. The generative method, search budget, number of tested sounds, and perceptual constraints can be kept the same while the source of guidance is changed. Such comparisons should include null controls in which neural guidance is disrupted while preserving the overall structure of the data, as well as non-neural objective controls in which the same generative and search procedures are guided by acoustic, perceptual, or other predefined objectives rather than brain-derived information. These controls allow researchers to determine whether discovered stimulus structures depend specifically on neural information or instead emerge from properties of the generative model, stimulus representation, or search procedure. These comparisons should be performed within the same independent human experiment, using new neural or behavioral measurements to determine whether brain-guided stimuli reveal structures that would not have been identified through conventional or non-neural search strategies. For example, if the goal is to locate a representational boundary, stimuli generated with brain guidance should reveal a clearer or more reproducible transition in new human measurements than stimuli obtained from a predefined or control search. If the goal is to distinguish competing explanations, brain-guided stimuli should produce a stronger and more reliable difference between the responses predicted by those explanations. If these advantages disappear when neural guidance is replaced by an appropriately matched alternative, the claim that brain-derived information itself provided additional scientific leverage would be weakened, even if the generative procedure still produced interesting sounds.

The comparisons described above require prespecified criteria for success, rather than judgments based on the novelty or perceptual quality of generated sounds. Three criteria are particularly important. First, the inferred brain–sound relationship should generalize to newly generated stimuli that were not used to estimate the mapping or guide the initial search, as assessed through independent neural measurements and, where appropriate, behavioral responses. Second, brain-guided selection should provide a measurable advantage over matched predefined or non-neural alternatives on a scientific outcome specified in advance. Depending on the research question, this may involve more reproducible localization of a representational boundary, more reliable identification of neural-response-defined stimulus organization, or stronger discrimination between competing explanations. Third, the advantage should be reproducible in independent measurements and robust to generator-specific artifacts or uncontrolled acoustic differences. For recursive experiments, success may additionally be reflected in improved out-of-sample prediction, reduced uncertainty about the target relationship, or increased stability of the inferred structure across successive iterations.

Practical implementation of brain-guided generation requires addressing a challenge that differs fundamentally from conventional stimulus-based experiments: neural measurements become part of the generative constraint itself. Consequently, measurement uncertainty is not only a limitation on statistical inference but can directly alter which regions of the stimulus space are explored and which sound structures are inferred as meaningful. Participant fatigue is therefore not merely a logistical concern; changes in attention, arousal, or task engagement across repeated generations may introduce state-dependent variability that is subsequently incorporated into the inferred brain–sound relationship. Likewise, the number of required trials is determined not only by estimating reliable responses, but by determining when the inferred stimulus organization becomes sufficiently stable to guide further generation. Adaptive sampling, active experimental design, and uncertainty-based stimulus selection provide potential solutions by concentrating measurements on stimulus regions where additional observations are expected to provide the greatest information gain [61,62]. In parallel, reliable estimation of stimulus-related neural structure will be critical, because uncertainty in neural measurements can directly distort the brain–sound relationships used to guide generation. Methods that account for neural variability and measurement noise will therefore be important for preventing spurious associations from being incorporated into generative constraints, particularly when exploring high-dimensional auditory spaces [63].

Generative models introduce a second set of practical challenges. Novel sounds produced by generative systems may contain artifacts or unintended acoustic regularities that are unrelated to the neural structure being investigated. Such artifacts could create misleading associations if generated stimuli are interpreted solely on the basis of model outputs. Future studies should therefore combine neural validation with independent acoustic characterization, behavioral evaluation, and comparisons against matched non-neural generation strategies to determine whether generated sounds preserve the intended stimulus relationships rather than merely reflect properties of the generative model. Specifically, this acoustic characterization should assess temporal discontinuities, rhythm or prosody where relevant, and source/scene coherence, rather than relying solely on spectral similarity to reference signals or generic perceptual-quality scores [5,34,48,49]. Similar concerns have motivated evaluation frameworks in generative modeling that emphasize generalization, controllability, and alignment between generated outputs and target representations rather than relying only on sample quality [64,65]. For brain-guided auditory generation, generated sounds must therefore be evaluated as scientific stimuli rather than solely as generative outputs.

With these evaluation requirements in view, the available evidence can be distinguished at three levels: a direct auditory demonstration, methodological capabilities established independently, and questions that remain unresolved for the integrated framework. At the first level, Xing et al. [24] synthesized sounds matched to brain activity and showed that these sounds recapitulated preferential neural responses to speech and music, with supporting behavioral evidence. This provides a direct auditory proof of principle, but does not establish the general reliability of brain–sound mappings or validate the full recursive framework proposed here.

Beyond this specific auditory demonstration, several methodological capabilities relevant to the framework have been demonstrated independently. Auditory encoding and neural–auditory representational alignment have been used to relate sound representations to human neural responses [20,21,33,40,41]; generative audio systems can produce controllable or novel auditory signals [22,23,42,43]; adaptive stimulus optimization has guided auditory experiments and other sequential searches [6,7,8,18,19,44,45]; and neural-guided generative stimulus exploration has been demonstrated in vision [14,15,16,17,29,46,47]. These studies provide methodological foundations for the proposed framework, but their individual successes do not establish that the methods can be integrated into a reliable auditory experimental process or that this integration will yield additional scientific value.

The central unresolved issue is therefore whether these demonstrated capabilities can be combined into a process that produces reliable and scientifically informative discoveries. As discussed above, this requires brain–sound mappings to generalize beyond the data used to estimate them [20,21,24,25,29,30,31,33], generated sounds to preserve the stimulus relationships relevant to the neural hypothesis without introducing confounding artifacts [4,28,64,65], and iterative experiments to remain reliable under practical constraints such as measurement noise, limited trial budgets, participant fatigue, and state-dependent variability [30,31,61,62,63]. Most importantly, it remains to be established whether repeated brain-guided generation and testing yield more reproducible or diagnostically informative scientific inferences than matched predefined or non-neural alternatives. Until these requirements are met, brain-guided generative auditory neuroscience should be regarded as a prospective research framework supported by a specific auditory proof of principle and independently demonstrated methodological capabilities, rather than as an already validated integrated system.

## 7. Limitations and Boundaries of Brain-Guided Discovery

Brain-guided generation can expand the space of auditory hypotheses, but it cannot adjudicate which of those hypotheses should ultimately be accepted as scientific explanations. Neural evidence can help formulate candidate hypotheses that would be difficult to derive from acoustic analysis alone, and generative systems can translate those hypotheses into sounds that can be tested experimentally. Yet neither a generated stimulus nor the response it is designed to elicit provides an autonomous verdict on what that result means. Determining whether a candidate explanation should be retained, revised, or rejected remains a human scientific responsibility, requiring investigators to judge the result in relation to competing explanations, experimental constraints, and the broader empirical context. Brain-guided generation should therefore be understood as a means of expanding and sharpening the hypotheses that can be brought into experiment, not as an autonomous process for deciding their scientific validity.

A second boundary concerns how the structures discovered through brain-guided generation should be interpreted. A generated stimulus space is not equivalent to the full space of auditory representations, because every discovery is constrained by the neural measurements, stimulus representations, and generative systems used to explore it. If a generator cannot produce a particular sound or variation, this does not imply that the auditory system does not encode that structure; it may simply indicate that the current generative space does not provide access to it. Conversely, an effective direction or feature combination within a generative representation should not be interpreted as a direct readout of how the brain organizes sounds. The discovered relationship may overlap with an underlying auditory organization within the range of stimuli that have been explored, while still providing only a partial description of the computations that generate the neural response. A correspondence that is useful for predicting or manipulating responses therefore does not necessarily reveal the complete structure of auditory representation. Determining its broader significance requires testing whether the inferred organization remains stable across alternative stimulus realizations, representations, and generative approaches. The goal of brain-guided discovery is therefore not to recover a definitive map of auditory space, but to identify experimentally useful structures whose relationship to human auditory organization can be progressively clarified through further testing.

Finally, recursive brain–sound inference requires a criterion for when continued exploration is no longer expected to substantially change the scientific inference. The endpoint of an adaptive loop should not be defined by reaching a maximal neural response or by exhausting the available stimulus space, because neither condition indicates that the underlying auditory organization has been resolved [18]. Instead, recursion should provide a principled way to reduce uncertainty about the relationship being investigated: additional generations can progressively refine candidate stimulus relationships, boundaries, or explanatory distinctions, while new human measurements can evaluate whether these inferred structures remain supported or whether alternative interpretations remain plausible [66,67]. This principle parallels stopping criteria in adaptive experimental design, where sampling is terminated not when optimization is complete but when additional observations provide limited expected information gain [18]. In brain-guided auditory experiments, once a candidate organization remains stable across independently generated stimuli, participants, or measurement conditions, the goal should shift from further adaptive exploration to confirmatory testing. The endpoint of brain-guided discovery is therefore not a sound that can no longer be improved, but a stimulus–brain relationship whose interpretation no longer depends on continued searching.

Beyond these epistemic and methodological boundaries, increasingly adaptive and individualized brain-guided experiments introduce additional requirements for reproducibility, model transparency, and responsible data management. A reproducible pipeline should document the complete process from neural preprocessing and target definition to auditory representation, brain–sound mapping, generative constraints, adaptive stimulus selection, and stopping criteria. Relevant implementation details include model architectures and versions, pretrained checkpoints where available, training-data provenance, key hyperparameters, random seeds, software dependencies, and stimulus-generation settings. Code, configurations, and stimulus metadata should be shared where feasible and consistent with participant consent, data-protection requirements, and model licensing [68]. When closed or proprietary models prevent full disclosure, these limitations should be stated explicitly, and the robustness of the findings should be assessed across available model versions or alternative generators. Such transparency is important because a result that depends strongly on one particular model or generation pipeline may not reflect a stable property of auditory representation.

Individualized neural models also raise privacy concerns because neural recordings and derived representations may contain or permit inferences about participant-specific characteristics [69]. Their collection, storage, reuse, and sharing should therefore be governed by informed consent, data minimization, appropriate access controls, and secure data management. A further methodological risk is adaptive overfitting: because each new stimulus is selected in response to previous neural measurements, chance fluctuations may be repeatedly reinforced and mistaken for meaningful brain–sound structure. This risk is related to the broader problem of circular analysis, in which selecting and evaluating effects using non-independent data can produce misleading conclusions [70]. Discovery and confirmation should therefore be separated through held-out stimuli, independent measurement sessions or participants where appropriate, and prespecified confirmatory outcomes. Shuffled or mismatched neural-guidance controls and stability checks across random seeds, model configurations, and generative pipelines can further help determine whether an inferred relationship reflects reproducible neural organization rather than measurement noise or model-specific idiosyncrasies. These safeguards are essential for establishing the credibility of increasingly adaptive and personalized auditory experiments.

## 8. Conclusions

Building on existing demonstrations of brain-guided sound construction and validation, the framework developed here extends the scientific use of brain-guided auditory generation in several connected directions. It organizes the scientific scope of the approach around six classes of auditory question for which relevant stimulus structure is not fully specified in advance, and develops recursive brain–sound inference as a way for new human measurements to refine the brain–sound relationship and guide subsequent experimental interventions. The framework also treats the added value of brain guidance as an empirical question, to be assessed against appropriately matched predefined or non-neural alternatives. These elements position brain-guided generative auditory neuroscience as a question-driven and iterative experimental strategy in which neural evidence helps shape the construction, testing, and progressive refinement of auditory experiments.

The strength of this approach will ultimately depend on whether brain guidance produces scientific evidence that cannot be obtained as effectively from predefined, non-neural, or otherwise matched alternatives. Brain-derived relationships can motivate candidate stimulus structures, and generative systems can instantiate them as experimental probes, but neither the generated stimuli nor the computational procedures that produce them determine their scientific meaning; interpretation remains tied to human experimentation, comparison, and inference. The central opportunity is therefore not to replace conventional auditory experimentation, but to add a complementary route in which neural evidence helps define the next experiment itself. If that route can reveal reproducible relationships that survive independent testing and remain informative beyond the particular models and generators through which they were discovered, brain-guided generation could become a powerful means of turning observations of auditory brain responses into progressively more targeted questions about human auditory organization.

## Figures and Tables

**Table 1:** Key concepts and their distinct roles in brain-guided generative auditory neuroscience.

Concept	Core Meaning	Key Distinction
Brain-guided generation	Using brain-derived information to guide sound generation and evaluation.	The overarching experimental framework.
Neural-guided stimulus optimization	Adjusting stimuli to target specific neural responses.	A specific stimulus-search or optimization procedure.
Adaptive experimental design	Selecting subsequent stimuli or measurements based on existing data.	A strategy for choosing what to test next.
Recursive brain–sound inference	Updating brain–sound relationships using new experimental measurements.	Emphasizes iterative refinement of the inferred mapping.

**Table 2:** Scientific questions, brain-guided generative strategies, and principal insights across the six opportunities proposed in Section 2.

Section/Scientific Opportunity	Core Scientific Question	Brain-Guided Generative Strategy	Principal Scientific Insight
2.1 Discriminative structure beyond predefined dimensions	What stimulus structure supports a known auditory distinction when the relevant dimensions are unknown?	Map the neural distinction onto a rich auditory space; Probe response-relevant directions or regions	Discriminative dimensions or feature combinations
2.2 Latent organization within predefined auditory classes	What latent organization exists within an established auditory class?	Infer neural clusters, gradients, or transitions; Generate within and across the inferred organization	Latent substructure beyond conventional labels
2.3 Representational invariance, equivalence, and boundaries	Which stimulus variations preserve a target neural representation, and where does equivalence break down?	Define a neural equivalence criterion; Probe equivalence regions and uncertain boundaries	Sharp or graded representational boundaries
2.4 Previously unspecified auditory groupings	Can neural–stimulus relationships reveal coherent sound groupings not specified in advance?	Identify neural-response-defined groupings; Generate within, around, and across inferred groupings	Reproducible neural-response-defined organization
2.5 Contextual relationships across auditory histories and scenes	Which contextual relationships systematically shape neural responses when relevant variables are unknown?	Map neural variation across histories and scenes; Generate or vary the inferred contextual relations	Generalizable contextual or relational dependencies
2.6 Individual- and state-dependent brain–sound relationships	Which brain–sound relationships are shared, individual-specific, or state-dependent?	Estimate listener-specific brain–sound mappings; Adapt exploration as internal state changes	Shared versus individualized auditory organization; State-dependent changes in sensitivity or engagement

## Data Availability

No new data were created or analyzed in this study. Data sharing is not applicable to this article.
